# Establishment of Transient Transformation Systems in Welsh Onion (*Allium fistulosum* L.): Hairy Root Induction and Protoplast Transformation

**DOI:** 10.3390/plants14172664

**Published:** 2025-08-26

**Authors:** Dan Wang, Yin Liu, Yao Zhang, Xiumei Huang, Jiaxuan Wang, Yi Wang, Yue Liu, Chao Yan, Bingsheng Lv, Yue Jia

**Affiliations:** 1 College of Horticulture, Qingdao Agricultural University, Qingdao 266109, China; wangdan199910@163.com (D.W.); zhangyao7923@163.com (Y.Z.); 18877584492@163.com (X.H.); 15997986051@163.com (J.W.); wangyi_2121@163.com (Y.W.); liuyue@qau.edu.cn (Y.L.); yanchao@qau.edu.cn (C.Y.); 2 College of Horticulture, Jilin Agricultural University, Changchun 130118, China; ly2941571760@163.com

**Keywords:** Welsh onion, transient transformation, hairy root induction, protoplast transformation

## Abstract

Welsh onion (*Allium fistulosum* L.), a globally significant vegetable, flavoring agent, and phytomedicine resource, has remained unavailable with established transient expression platforms for functional genomic investigations. To address this critical methodological limitation, we present systematically optimized protocols for both *Agrobacterium*-mediated hairy root transformation and protoplast transient expression systems, achieving significant advances in transformation efficiency for this species. Through systematic optimization of key parameters, including *Agrobacterium rhizogenes* (*A. rhizogenes*) strain selection (with Ar.Qual demonstrating superior performance), explant type efficacy, bacterial suspension optical density (OD_600_ = 0.3), and acetosyringone induction concentration (100 μM), we established a highly efficient stem disc infection methodology, achieving 88.75% hairy root induction efficiency. Subsequent optimization of protoplast isolation protocols identified the optimal enzymatic digestion conditions: 6-h dark digestion of young leaves using 1.0% (*w*/*v*) Cellulase R-10, 0.7% (*w*/*v*) Macerozyme R-10, and 0.4 M mannitol, yielding 3.3 × 10^6^ viable protoplasts g^−1^ FW with 90% viability. System functionality validation through PEG-mediated transient transformation demonstrated successful green fluorescent protein (GFP) reporter gene expression, confirmed by fluorescence microscopy. As the first documented transient expression platforms for Welsh onion, these protocols enable essential molecular investigations, including in planta promoter activity profiling, subcellular protein localization, and CRISPR-based genome-editing validation. This methodological breakthrough overcomes previous technical constraints in Welsh onion molecular biology, providing critical tools for accelerated gene functional characterization in this agriculturally important species.

## 1. Introduction

Welsh onion (*Allium fistulosum* L.), a biennial plant of the genus Allium (family Amaryllidaceae), originated in northwest China and was domesticated there before spreading to Japan, Europe, and the Americas via maritime migration routes [1]. As a globally important pungent vegetable, Welsh onion is widely cultivated and traded, prized for its unique flavor, nutritional value, and medicinal properties [2]. It contains bioactive compounds such as pectin, allicin, and selenium, which contribute to cholesterol reduction, vasodilation, and inhibition of cancer cell growth [3,4,5]. With increasing demand for nutritious and health-promoting foods, Welsh onion holds significant potential in international markets. However, genomic research on Welsh onion has been hindered by its enormous genome (~12 Gb), which is four times the size of the human genome and classified as a “super-genome.” This complexity delayed genome sequencing and assembly until recent breakthroughs in 2022 and 2023, which produced chromosomal-level genomes for Welsh onion, garlic, and onion, enabling functional genomics studies [6,7]. In Welsh onion, wax synthesis and metabolism-related genes were explored, and male sterility-related ATPase isoenzyme and *atpA* gene were comparatively analyzed [8,9,10]. Despite these advances, research progress remains slow due to the lack of a stable genetic transformation system. To address this challenge, establishing an efficient transient transformation system is critical for gene function analysis.

Transient gene expression is a powerful tool for studying gene function in plants. This technology introduces exogenous genes into host cells via expression vectors, enabling rapid high-level expression or silencing without genomic integration. Although transiently expressed genes are not heritable, this approach offers a short experimental cycle, low risk of gene drift, and high biosafety, making it indispensable for functional genomics [11]. Since its development in the 1960s, common transient transformation methods include *Agrobacterium*-mediated transformation, PEG-mediated protoplast transfection, plant virus vectors, and gene gun bombardment [12].

The *Agrobacterium*-mediated transient expression system is particularly efficient for horticultural plants. In this method, exogenous genes are cloned into binary vectors, transformed into *A. rhizogenes*, and delivered into plant tissues via injection, vacuum infiltration, or other infection techniques. Key factors affecting efficiency include bacterial strain selection, concentration, plant growth conditions, and tissue type [13]. For example, in jute, optimized hairy root induction using *A. rhizogenes* K599 enabled functional studies via overexpression, subcellular localization, and protein interaction assays [14]. In soybean hairy roots derived from green cotyledons, overexpression of *GmFUS3* (with *GUS* as a control gene) preferentially increased triacylglycerol (TAG) production enriched in linoleic and linolenic acids while reducing oleic acid levels [15]. The root hair system can rapidly test the editing efficiency in horticultural plants, which is an important application of the root hair system [16].

Protoplasts are another essential tool in plant biotechnology, serving as versatile platforms for protein function analysis [17,18,19], cell signaling studies [20,21,22], gene expression regulation [23,24], cell biological processes [25], and CRISPR/Cas9 genome editing [26]. In some species, protoplasts can even regenerate into whole plants, accelerating trait improvement [27,28,29]. In monocot research, a dual-fluorescence reporting system has been established in rice protoplasts that enables quantitative detection of intracellular ribosome stalling at single-cell resolution. This system provided robust evidence elucidating how TMS5 regulates thermosensitive male sterility in rice [30]. Separately, wheat studies utilizing protoplast transformation indicate that MHS1.1 and MHS2.2 act as core regulatory elements for *Pm21* expression, playing a key role in powdery mildew resistance [31]. Recently, an efficient protoplast regeneration system was developed for *Salvia miltiorrhiza* Bunge. Using this system, researchers achieved highly efficient editing of multiple transcription factor genes involved in bioactive compound biosynthesis by single-transfection delivery of non-transgenic CRISPR/Cas9 reagents into protoplasts, resulting in biallelic edits. Crucially, stable knockout plants were regenerated via this novel system. This technological breakthrough offers a new strategy for enhancing bioactive compound content in heterozygous, genetically recalcitrant medicinal plants [32]. However, protoplast isolation remains challenging in many species, including Welsh onion, due to variability in cell wall composition. Factors such as enzyme mixture, osmoticum concentration, incubation time, and temperature critically influence protoplast yield and viability [33,34,35,36].

In this study, we established two transient transformation systems for Welsh onion. For hairy root induction, we optimized parameters such as *A. rhizogenes* strain, infection site, bacterial density (OD_600_), and acetosyringone (AS) concentration to develop a simple and efficient hairy root system. For protoplast transformation expression, we evaluated enzyme composition, mannitol concentration, and digestion time to achieve high-yield, high-viability protoplasts. Using PEG-mediated transfection, we successfully expressed GFP in Welsh onion protoplasts, validating the system’s feasibility. To our knowledge, this is the first report of a transient transformation system in Welsh onion. These methods will facilitate gene functional studies and breeding applications in this economically important crop.

## 2. Materials and Methods

### 2.1. Establishment of Hairy Root Induction System

#### 2.1.1. Aseptic Seedling Culture and the Acquisition of Explants

The study utilized seeds of the ‘Zhangqiu Dawutong’ Welsh onion variety provided by the College of Horticulture at Qingdao Agricultural University as experimental material. For seed sterilization, 3 g of seeds was placed in a 50 mL conical flask containing 35 mL of 1% sodium hypochlorite solution with a magnetic stirrer rotor, then stirred at 100 r/min for 3 h. The seeds were transferred to a sterile workbench and washed three times with sterile water, followed by immersion in 75% ethanol for 3 min and three additional sterile water washes. Subsequent sterilization involved soaking in 2% sodium hypochlorite solution for 20 min, followed by six sterile water washes. After removing excess moisture with sterile filter paper, the sterilized seeds were placed in a sterile Petri dish lined with filter paper to complete disinfection. The surface-sterilized seeds were then inoculated onto MS medium supplemented with 4 g/L Gelrite as a gelling agent and initially grown in darkness at 24 °C for 14 d before transferring to light conditions for another 14 d to obtain axenic seedlings. Using a sterile scalpel, roots, stems, and leaves from these sterile seedlings were excised into 0.5–1 cm segments, with the root–stem junction region (0.5–1 cm in length) specifically designated as stem tissue for experimental purposes.

#### 2.1.2. Plasmid and Bacterial Strains

The study employed the pCAMBIA1305.4-GFP plasmid carrying the green fluorescent protein gene for the transfection of *A. rhizogenes* strains K599, LBA9402, and Ar.Qual, all of which were procured from Qingdao Zhuochuang Laboratory Service Co., Ltd. (Qingdao, China). and maintained in 50% glycerol stock solutions at −80 °C for long-term storage.

#### 2.1.3. Reagents

MS Medium (4.43 g/L MS basal salts, 30 g/L sucrose, 4 g/L Gelrite, pH 5.8);

Infection Solution (MS basal medium supplemented with 2.5 mM MES and 100 μM acetosyringone, pH 5.8);

Co-culture Medium (MS basal medium containing 2.5 mM MES, 100/200/300 μM acetosyringone, and 4 g/L Gelrite, pH 5.8);

Screening Medium (MS basal medium with 300 mg/L timentin, 100 mg/L kanamycin, and 4.5 g/L Gelrite, pH 5.8);

LB Liquid Medium (5 g/L yeast extract, 10 g/L tryptone, 10 g/L NaCl).

#### 2.1.4. *A. rhizogenes*-Mediated Hairy Root Induction

The *A. rhizogenes* strain carrying the pCAMBIA1305.4-GFP plasmid was streaked onto LB solid medium supplemented with 100 mg/L kanamycin and incubated inverted at 28 °C in darkness for 24 h, with this activation process repeated once. Following the second activation, 5 mL of infection solution (pre-supplemented with acetosyringone) was added to the plate, and the bacterial cells were gently resuspended using a 1 mL pipette. The resulting suspension was transferred to a sterile 50 mL centrifuge tube and diluted with fresh infection solution to achieve an OD_600_ between 0.3 and 0.8. Welsh onion root, stem, and leaf explants (0.5–1 cm segments) were immersed in this bacterial suspension for 15–20 min, then blotted dry on sterile filter paper before being placed on co-cultivation medium overlaid with filter paper. After 3 d of dark incubation at 23 °C, the explants underwent eight washes with sterile water containing 300 mg/L cefotaxime until the wash solution appeared clear. Following final blotting on sterile filter paper and complete air-drying, the explants were transferred to selection medium and cultured under ambient light conditions for 14 d. GFP-positive transgenic hairy roots could be identified using a LUYOR-3280 GFP visualization flashlight, with transformation efficiency calculated as

(1) $$\left(number\;of\;explants\;producing\;GFP\;-\;positive\frac{roots}{total}number\;of\;explants\right)\times 100\%$$

(2) $$Transformation\;efficiency=\frac{Number\;of\;explants\;producing\;GFP-positive\;roots}{Total\;number\;of\;explants}\times 100\%$$

#### 2.1.5. PCR Verification Experiment for *GFP* Transgene Integration

In the PCR verification experiment for *GFP* transgene integration, DNA extraction was performed using the CTAB (Cetyltrimethylammonium Bromide) method. The control group used DNA from control roots induced by *A. rhizogenes* Ar.Qual as the material, while the experimental group used DNA from independent transgenic hairy root systems. The primers were designed as follows: F: tcgtgaccaccttcacctac, R: tgtcgacggtatcgataagc. In order to amplify the *GFP* gene sequence, PCR was initiated by a hot start at 94 °C for 10 min and amplified during 30 cycles at 94 °C for 30 s, 53 °C for 30 s, and 72 °C for 90 s, followed by a final extension step at 72 °C for 5 min. The electrophoresis of the PCR products was performed on 1.2% agarose gel under a constant voltage of 80 V. The gel was subsequently stained with ethidium bromide solution and examined under UV light. TIANGEN Marker III DNA Ladder (Tiangen Biotech, Beijing, China) was employed as the molecular size standard.

### 2.2. Establishment and Optimization of Protoplast Transformation

#### 2.2.1. Plant Materials and Growth Conditions

The study employed seeds of the ‘Zhangqiu Dawutong’ Welsh onion cultivar provided by Qingdao Agricultural University’s College of Horticulture, which were uniformly sown in a 3:1 (*w*/*w*) mixture of nutrient soil and vermiculite and cultivated in a growth chamber maintained at 23 °C with a 12 h photoperiod.

#### 2.2.2. Plasmid Preparation

The *Escherichia coli* DB3.1 strain harboring the pAL902-GFP plasmid was kindly provided by Qingdao Agricultural University’s College of Life Science. Plasmid extraction was performed using the EndoFree Maxi Plasmid Kit (TIANGEN, Beijing, China), with quality control parameters requiring a minimum DNA concentration of 1 µg/µL and an OD_260_/OD_280_ ratio between 1.8 and 2.0, followed by storage at −20 °C for long-term preservation.

#### 2.2.3. Protoplast Isolation

Two-week-old Welsh onion seedlings were selected, rinsed with double-distilled water (ddH_2_O), blotted dry with absorbent paper, and placed in Petri dishes for surface drying, with only leaves used for protoplast isolation. Approximately 0.5 g of leaf tissue was rapidly sliced into 0.5–1.0 mm fragments using a sharp double-sided razor blade and immediately immersed in 10 mL of enzyme solution containing 20 mM KCl (Shanghai Aladdin Biochemical Technology), 20 mM MES-KOH (pH 5.8), 10 mM CaCl_2_, 1.0 g/L bovine serum albumin, 0.5–1.5% (*w*/*v*) Cellulase R-10 (Yakult Honsha), 0.3–0.7% (*w*/*v*) Macerozyme R-10 (Yakult Honsha), and 0.3–0.6 M mannitol (all from Qingdao Zhuochuang Laboratory unless specified). Following 30 min vacuum infiltration (−0.1 MPa) at 26 °C in darkness, enzymatic digestion proceeded at 50 r/min for 4–10 h until complete protoplast release. The digestion was terminated by adding an equal volume of ice-cold W5 buffer (154 mM NaCl, 4 mM MES, 125 mM CaCl_2_, 5 mM KCl, pH 5.7), followed by filtration through a 70 μm nylon mesh to remove undigested debris. The filtrate was collected in 50 mL round-bottom tubes and centrifuged at 100 r/min for 3 min (Eppendorf Centrifuge 5920 R, Hamburg, Germany) to pellet protoplasts, after which the supernatant was carefully aspirated to obtain purified protoplast preparations.

#### 2.2.4. Protoplast Counting and Viability Assessment

Protoplast quantification was performed using a dual-chamber hemocytometer (XB.K.25; QiuJing, Shanghai, China) under fluorescence microscopy. For viability assessment, 100 μL of protoplast suspension was mixed with 1 μL of 0.02% fluorescein diacetate (FDA; Shanghai Aladdin Biochemical Technology, Shanghai, China) and incubated for 5 min in darkness, followed by microscopic examination of 20 μL aliquots under positive fluorescence (Leica DM2500/DM2500 LED, Weztlar, Germany). Viable protoplasts exhibiting green fluorescence were enumerated, with three biological replicates per sample. Protoplast yield was calculated as

(3) $$Y=n\times 10^{4}\times p/g\;FW$$

where Y represents yield (protoplasts/g fresh weight), n is protoplast concentration (protoplasts/mL), p is total suspension volume (mL), and g FW is fresh leaf weight. Viability percentage was determined as (fluorescent protoplasts per field/total protoplasts per field) × 100.

(4) $$Protoplast\;yield=Protoplast\;concentration\times 10^{4}\times Total\;suspension\;volume÷Fresh\;leaf\;weight$$

(5) $$Viability\;percentage=\frac{Fluorescent\;protoplasts\;per\;field}{Total\;protoplasts\;per\;field}\times 100\%$$

#### 2.2.5. PEG-Mediated Protoplast Transfection

The harvested protoplast pellet was resuspended in 5 mL of ice-cold W5 solution and maintained on ice for 30 min under light-protected conditions. After discarding the supernatant, the protoplasts were resuspended in 1 mL of MMG solution (0.4 M mannitol, 15 mM MgCl_2_, 4 mM MES, pH 5.7). For transformation, 200 µL of the protoplast suspension was gently combined with 20 µL (1 µg/µL) of pAL902-GFP plasmid in a 2 mL round-bottom centrifuge tube, followed by the addition of an equal volume (200 µL) of PEG solution (30% PEG4000, 0.4 M mannitol, 0.1 M CaCl_2_). The mixture was incubated at 24 °C for 20 min in darkness. The transformation was terminated by adding 1 mL of W5 buffer, followed by centrifugation at 100 r/min for 2 min. After supernatant removal, the transformed protoplasts were washed with 1 mL of W5 buffer and transferred to a 35 mm cell culture dish for overnight incubation at 24 °C in complete darkness. GFP expression in transformed protoplasts was verified using epifluorescence microscopy after 20 h of incubation.

## 3. Results

### 3.1. Establishment of Hairy Root Induction System

#### 3.1.1. Optimizing Adventitious Root Induction in Welsh Onion: Evaluating *A. rhizogenes* Strains and Explant Selection

**Figure 1:**
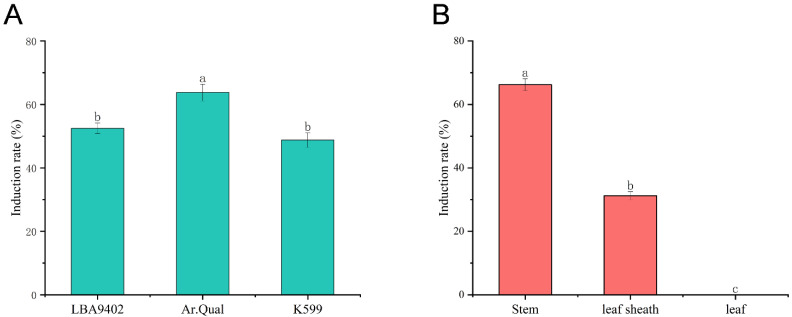
Differential effects of *A. rhizogenes* strains. (**A**) Induction efficiency of three tested strains (LBA9402, Ar.Qual, and K599) when infecting stem explants. (**B**) Hairy root induction efficiency in different explant types (stems, leaf sheaths, and leaves). Different lowercase letters above bars denote statistically significant differences (*p* < 0.05) according to one-way ANOVA with Tukey’s post hoc test.

The induction efficiency of hairy roots in Welsh onion was significantly influenced by both *A. rhizogenes* strains and explant types. Among the three tested strains (LBA9402, Ar.Qual, and K599), Ar.Qual demonstrated the highest induction efficiency (66.25%) when infecting stem explants (Figure 1A).

Subsequent comparative experiments using Ar.Qual with different explant types (stems, leaf sheaths, and leaves) revealed that the stems exhibited greater susceptibility to *A. rhizogenes* infection compared to other tissues, showing the highest hairy root induction rate, while leaves failed to produce any hairy roots. These results collectively demonstrate that Ar.Qual is the optimal *A. rhizogenes* strain for Welsh onion hairy root induction, with stem tissue serving as the most effective explant material (Figure 1B).

#### 3.1.2. Optimization of Induction Conditions for Hairy Roots

To optimize hairy root induction efficiency, infection conditions were systematically evaluated using *A. rhizogenes* Ar.Qual-infected Welsh onion stems through orthogonal experiments testing OD_600_ values and acetosyringone (AS) concentrations. Under optimal conditions (OD_600_ = 0.3, 100 μM AS), maximal induction efficiency reached 88.75%. (Table 1).

For the purpose of establishing the genetic transformation system of the Welsh onion hairy root induction system, *A. rhizogenes* Ar.Qual harboring the plasmid p35S-*GFP* was transformed into Welsh onion hairy roots. The transformation protocol includes the following main steps: pre-cultivation of explants and preparation of Ar.Qual, infection, induction of transgenic hairy roots, and subculturing hairy roots. Briefly, stem segment explants were prepared by complete root removal and immersed in either *A. rhizogenes* Ar.Qual harboring a GFP-containing vector or *A. rhizogenes* Ar.Qual without the *GFP* vector (as negative control). After co-cultivation, explants were subsequently introduced to the decontamination medium.

**Figure 2:**
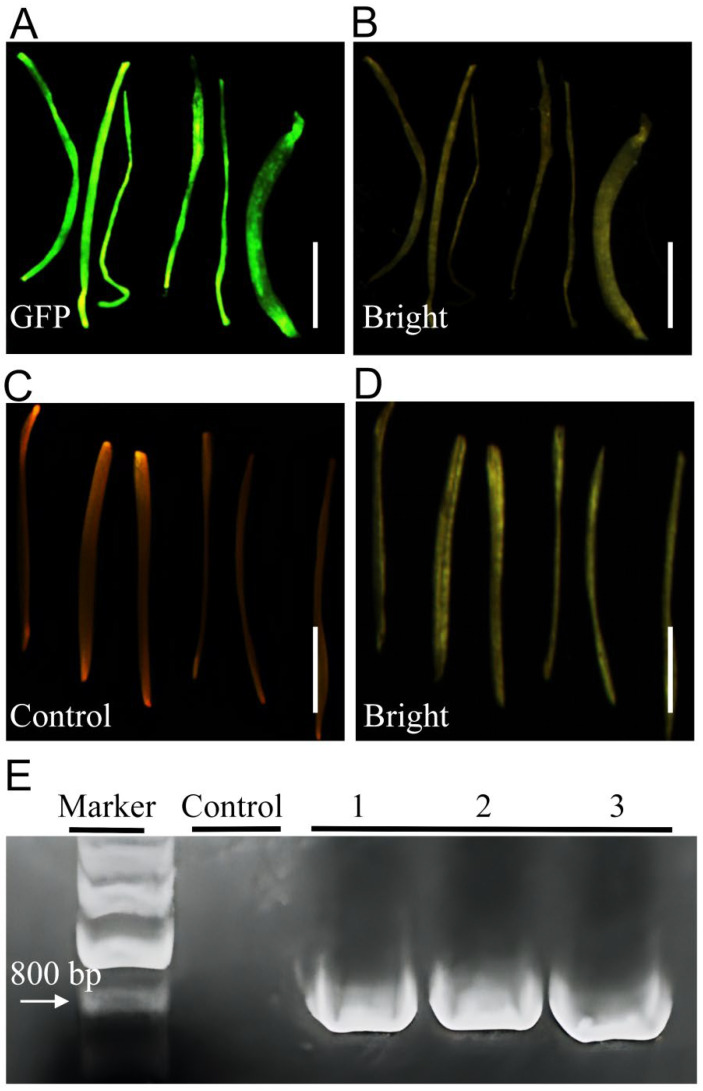
Hairy root induction in Welsh onion. (**A**) *GFP*-positive hairy roots excised from stem segments following induction and liquid suspension culture (fluorescent view). (**B**) Bright-field image corresponding to (**A**). (**C**) Non-transformed control roots induced by *A. rhizogenes* Ar.Qual (fluorescent view). (**D**) Bright-field image corresponding to (**C**). (**E**) PCR verification of GFP transgene integration. (Marker: DNA Marker, Control: Negative control, Numbers 1–3: independent transgenic hairy root lines. Bar = 2 mm.)

The GFP signal became detectable approximately 2 weeks after inoculation. Positively transformed hairy roots could then be excised and transferred to liquid suspension culture to facilitate expansion of *GFP*-positive roots (Figure 2A–D). Molecular confirmation of transgenic hairy roots was performed via PCR amplification of the *GFP* gene fragment (Figure 2E). The successful amplification of *GFP* gene fragments from transgenic hairy roots confirmed the actual transfer of foreign genes into hairy roots of Welsh onion. Collectively, our experiments demonstrate that the optimized *A. rhizogenes*-mediated transient transformation protocol efficiently generates putative hairy roots in Welsh onion with rapid transgene expression (e.g., *GFP*).

### 3.2. Establishment of the Protoplast Transformation System

#### 3.2.1. Determination of the Optimum Enzyme Concentration for the Isolation of Protoplasts from Welsh Onion Leaves

Enzyme concentration critically impacts protoplast isolation efficiency and viability. To determine the optimal enzymatic digestion conditions, we employed an orthogonal experimental design to systematically evaluate nine distinct concentration combinations of cellulase R-10 and macerozyme R-10 (Table 2).

Optimal protoplast isolation was achieved using 1.0% (*w*/*v*) Cellulase R-10 and 0.7% (*w*/*v*) Macerozyme R-10, demonstrating superior separation efficiency among all treatments (Table 2). Treatment 6 yielded the highest protoplast quantity (1.84 × 10^6^ protoplasts g^−1^ FW) with 89.67% viability. Polar analysis revealed Macerozyme R-10 exerted significantly greater influence on protoplast yield than Cellulase R-10 (*p* < 0.05), indicating its predominant role in facilitating efficient protoplast release from Welsh onion leaf tissues. These results establish the 1.0% Cellulase R-10/0.7% Macerozyme R-10 combination as the optimal enzymatic formulation for Welsh onion protoplast isolation.

#### 3.2.2. Determination of Optimum Mannitol Concentration and Enzyme Digestion Time for Protoplast Isolation from Welsh Onion Leaves

**Figure 3:**
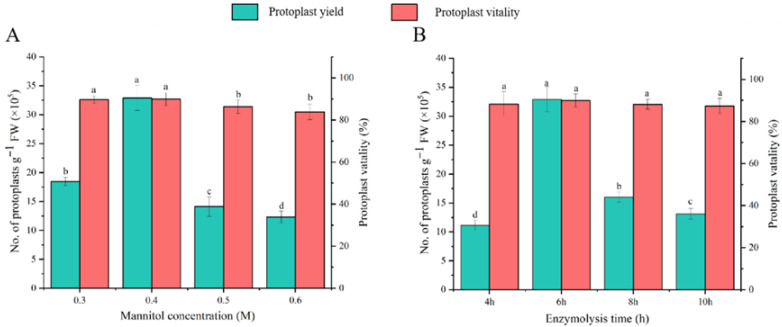
Effects of mannitol concentration (**A**) and enzymatic digestion duration (**B**) on protoplast yield and viability in Welsh onion (*Allium fistulosum* L.) leaves. Different lowercase letters in the notes indicate significant differences among treatments at *p* < 0.05 level.

Previous studies have demonstrated the significant influence of mannitol concentration and enzymatic digestion duration on protoplast isolation efficiency [33] Building upon our established optimal enzyme concentrations (1.0% Cellulase R-10 and 0.7% Macerozyme R-10), we systematically evaluated mannitol concentrations (0.3–0.6 mol/L) and digestion times (4–10 h) through one-way ANOVA to determine optimal isolation conditions for Welsh onion leaf protoplasts. Protoplast yield and viability assessments following 6 h digestions revealed concentration-dependent effects, with 0.4 mol/L mannitol yielding peak results (3.3 × 10^6^ protoplasts g^−1^ FW, 90% viability) (Figure 3B). Subsequent increases in mannitol concentration adversely affected both parameters. Temporal analysis showed maximal yield at 6 h (3.3 × 10^6^ protoplasts g^−1^ FW), decreasing significantly to 1.3 × 10^6^ protoplasts g FW^−1^ with prolonged digestion, while viability remained statistically unchanged (*p* > 0.05) (Figure 3B). These findings establish 0.4 mol/L mannitol and 6 h digestion as optimal conditions for Welsh onion protoplast isolation.

In previous reports, mannitol concentration and enzyme digestion time significantly affected the isolation efficiency of protoplasts. We further explored the mannitol concentrations (0.3, 0.4, 0.5, and 0.6 mol/L) and enzyme digestion times (4, 6, 8, and 10 h) by a one-way analysis to determine the optimal conditions for isolating protoplasts from the leaves of Welsh onion, on the basis of the optimal concentrations of Cellulase R-10 and Macerozyme R-10 previously determined.

In the enzymatic solution containing 1.0% Cellulase R-10 (*w*/*v*) and 0.7% Macerozyme R-10 (*w*/*v*), different concentrations of mannitol were added, and the production and viability of protoplasts were counted after 6 h of enzymatic digestion. Significantly, the yield of protoplasts first increased with the gradient change in mannitol concentration, and peaked at 3.3 × 10^6^ protoplasts g^−1^ FW and 90% activity when 0.4 mol/L mannitol was added. A further increase in mannitol concentration resulted in a decrease in yield and activity (Figure 3A). Therefore, 0.4 mol/L mannitol was considered to be the optimal concentration for the isolation of protoplasts from Welsh onion leaves.

Next, we further explored the enzymatic digestion time. As the duration of enzyme digestion increased, the protoplast yield first increased and peaked at 6 h (3.3 × 10^6^ protoplasts g^−1^ FW). Subsequently, the yield decreased to 1.3 × 10^6^ protoplasts g^−1^ FW, demonstrating a significant difference in the yield, but no significant difference in the protoplast viability (Figure 3B). These results indicate that 6 h is the optimal treatment time for isolating protoplasts from Welsh onion leaves.

**Figure 4:**
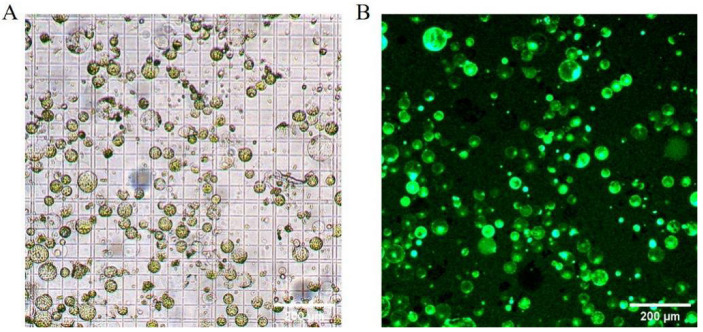
Morphological characterization of Welsh onion (*Allium fistulosum* L.) leaf protoplasts isolated under optimized enzymatic conditions. (**A**) Bright-field microscopy of purified protoplasts. (**B**) Viability assessment using fluorescein diacetate (FDA) staining. Scale bars: 200 µm.

The optimized protocol for high-yield protoplast isolation from Welsh onion leaves employs a 6 h enzymatic digestion using a solution containing 1.0% (*w*/*v*) Cellulase R-10, 0.7% (*w*/*v*) Macerozyme R-10, and 0.4 M mannitol. This standardized method consistently yields protoplasts with both high quantity (3.3 × 10^6^ protoplasts g FW^−1^) and viability (90%), as demonstrated in Figure 4. The resulting protoplast preparations exhibit sufficient quality for downstream transformation applications, including transient gene expression studies.

#### 3.2.3. PEG-Mediated Transient Transformation of Welsh Onion Leaf Protoplasts

**Figure 5:**
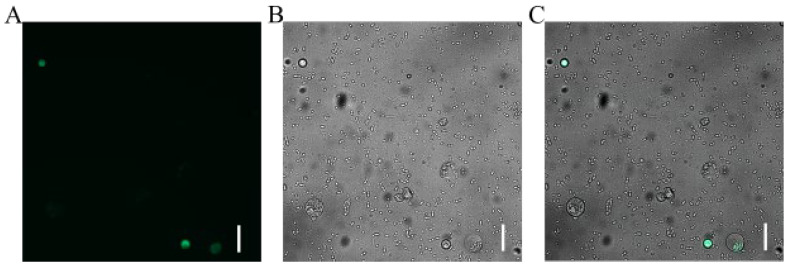
Transient GFP expression in Welsh onion leaf protoplasts visualized by fluorescence microscopy. (**A**) Bright-field image showing protoplast morphology. (**B**) GFP fluorescence signal (excitation/emission: 488/507 nm). (**C**) Merged image confirming successful transformation. Scale bar: 100 μm.

We successfully established a PEG-mediated transient transformation system for Welsh onion leaf protoplasts using the pAL902-GFP plasmid. The transformation protocol involved mixing 20 μL of plasmid DNA (1 μg/μL) with 200 μL of protoplast suspension in the presence of 30% PEG 4000, followed by 20 min incubation in darkness. After 20 h of culture under optimal conditions, distinct GFP fluorescence signals were detected via fluorescence microscopy (Figure 5), confirming successful transgene expression. Quantitative analysis of three independent biological replicates demonstrated a transformation efficiency of 10.47%.

**Figure 6:**
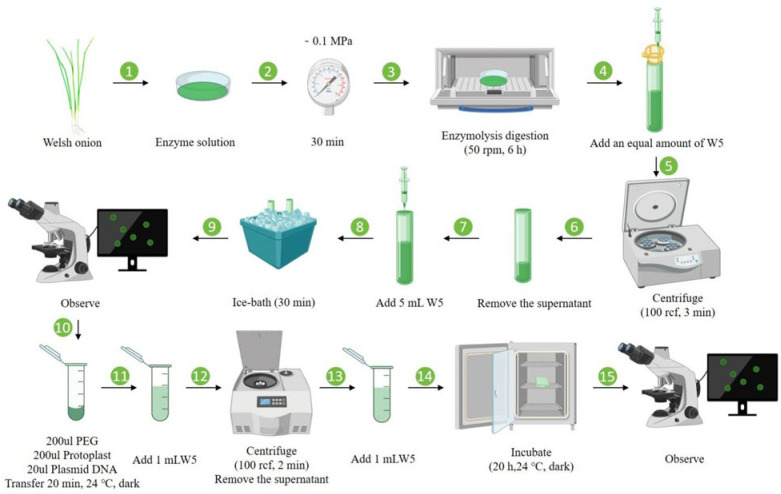
Schematic workflow for protoplast isolation, purification, and transient transformation from Welsh onion leaves. The optimized protocol comprises (1–3) enzymatic digestion (1.0% Cellulase R-10, 0.7% Macerozyme R-10, 0.4 M mannitol, 6 h); (4–9) purification through sequential filtration and centrifugation; (10–15) PEG-mediated transformation (30% PEG4000, 20 min) yielding viable protoplasts suitable for transient gene expression studies.

Figure 6 summarizes the optimized workflow for protoplast isolation, purification, and transient transformation in Welsh onion, where two-week-old leaf tissues were finely chopped and digested in enzymatic solution (1.0% Cellulase R-10, 0.7% Macerozyme R-10, 0.4 M mannitol) for 6 h at 26 °C, followed by sequential purification through filtration (70 μm nylon mesh) and centrifugation (100 r/min, 3 min) to obtain viable protoplasts, which were subsequently transformed via a PEG-mediated method (30% PEG4000, 20 min incubation) and successfully expressed GFP, as confirmed by fluorescence microscopy, demonstrating an average transformation efficiency of 10.47% across three biological replicates.

## 4. Discussion

The development of transient expression systems has significantly facilitated genetic engineering research by reducing experimental duration and costs [37]. Establishing an efficient hairy root induction system represents a fundamental requirement for superior monoclonal selection. As root induction conditions vary substantially among plant species without established universal protocols, continuous optimization through empirical investigation remains essential for identifying species-specific optimal conditions [38]. It should be noted that while our transient transformation system enables rapid gene validation (e.g., *GFP* expression in 4–6 weeks), long-term stability remains unverified. Future bioproduction applications will require validation through extended subculturing (>6 months) to confirm genomic stability and biosynthetic consistency. This optimized protocol nevertheless provides an efficient preliminary screening platform for functional studies prior to stable line development.

Strain specificity exerts profound effects on transformation efficiency, with significant variations observed both across plant species and among different tissues within the same plant. Commonly employed *A. rhizogenes* strains include Ar.Qual, K599, A4, ATCC15834, and LBA9402. Comparative studies in tea plants demonstrated that ATCC15834-mediated transformation efficiency exceeded that of *A. rhizogenes* K599 by 3.5-fold (bacterial soaking) and 8.9-fold (vacuum infiltration) [39]. Conversely, in pea plants, K599 induced more numerous and longer hairy roots than Ar.Qual [40].

Bacterial concentration significantly influences hairy root induction [41]. During wound healing, host plants release phenolic compounds like acetosyringone (AS) that activate *A. rhizogenes* virulence mechanisms. Pre-cultivation enhances this signaling molecule production, thereby improving transformation rates. Our orthogonal experiments identified optimal induction conditions for Welsh onion as OD_600_ = 0.3 and AS concentration = 100 μM [42].

Protoplasts, defined as plant cells with intact plasma membranes following cell wall removal, retain full cellular viability [43]. Their wall-less nature facilitates efficient uptake of genetic material (nuclear DNA, organellar genomes, or exogenous DNA fragments), making them ideal for genetic manipulation [44]. Protoplast isolation represents a critical step in plant cell biology studies, with multiple factors influencing isolation efficiency. Selection of source material primarily determines protoplast yield and viability [45]. Although both leaf tissue and callus can serve as protoplast sources, callus-derived protoplasts present several limitations: their colorless, transparent nature complicates handling and subsequent transformation, and their undifferentiated state restricts applicability for certain biological studies. In contrast, young leaves (2 weeks old in this study) offer superior material due to easy accessibility, loose cellular organization, and minimal harvest damage.

Enzyme selection and concentration critically affect protoplast isolation efficiency. The synergistic combination of cellulase (cellulose degradation) and macerozyme (pectin and hemicellulose breakdown) typically proves most effective. For tea leaves, 1.5% Cellulase R-10 with 0.4–0.6% Macerozyme R-10 yielded maximum protoplast production (3.27 × 10^6^ protoplasts g^−1^ FW) and viability (92.94%) [46]. Optimal enzyme concentrations vary by species and tissue type: carnation protoplast isolation required 1.0% Cellulase R-10 and 0.1% Macerozyme R-10 [47], while Uncaria rhynchophylla leaf tissue showed best results with 1.25% Cellulase R-10 and 0.6% Macerozyme R-10 (yield: 1.5 × 10^7^ protoplasts g^−1^ FW; viability > 90%) [48]. Our orthogonal tests identified optimal enzyme concentrations for Welsh onion, noting that excessive enzyme concentrations reduced yields, likely through plasma membrane damage.

Osmotic stabilization represents another critical parameter, as hypotonic conditions can induce protoplast lysis [49]. Mannitol and sorbitol serve as conventional osmotic stabilizers in protoplast isolation systems. We observed that Welsh onion protoplast yield and viability declined with increasing mannitol concentrations above 0.4 M, which provided optimal osmotic conditions.

Enzymatic digestion duration significantly impacts isolation efficiency [50]. Both insufficient and excessive digestion prove detrimental—our results identified 6 h as optimal for Welsh onion, balancing complete cell wall digestion against membrane integrity preservation.

The absence of cell walls makes protoplasts particularly suitable for transient transformation studies, enabling efficient exogenous DNA uptake. Primary transformation methods include PEG-mediated transfection, electroporation, and microinjection [51]. Recently, *AtGRF5* has been used to enhance genetic transformation efficiency in cucurbit crops [52]. This strategy can also be used in promoting transient transformation efficiency in the future.

In this study, we achieved successful PEG-mediated transformation of Welsh onion protoplasts using pAL902-GFP (10.47% efficiency), validating their suitability for molecular studies. While these initial results establish a foundational protocol, transformation efficiency requires further optimization through parameter adjustment (PEG type/concentration, plasmid DNA concentration, incubation time). For instance, kiwifruit protoplast transformation reached 43.5% efficiency using 40% PEG4000, 15 min incubation, and 10–15 ng/μL plasmid DNA with 24–26 h culture [53]. While the achieved PEG-mediated transformation efficiency (10.47%) enables preliminary gene screening, we propose three tractable strategies for enhancement: vector optimization: testing monocot-optimized promoters (e.g., maize *Ubi1*) and nuclear localization signal (NLS) tags to improve DNA nuclear delivery; PEG protocol refinement: titrating PEG4000 concentration (beyond 20–40%) with reduced exposure time (<10 min) to mitigate cytotoxicity; nuclease inhibition: adding carrier DNA (e.g., salmon sperm DNA) during transfection to competitively bind nucleases. Additional investigation of Welsh onion protoplast transformation conditions remains warranted.

## 5. Conclusions

In this study, we successfully established two efficient transient transformation systems for Welsh onion. For hairy root induction, we systematically optimized critical parameters, including (1) *A. rhizogenes* strain selection, (2) explant type, (3) bacterial suspension density (OD_600_), and (4) acetosyringone (AS) concentration in co-culture medium. Our results demonstrated that infection of stem discs with *A. rhizogenes* Ar.Qual at OD_600_ = 0.3 in medium containing 100 μM AS achieved maximal induction efficiency of 88.75%.

Regarding protoplast transient transformation, we developed an optimized isolation protocol for Welsh onion leaf protoplasts. Through systematic evaluation of key factors—(1) enzyme composition, (2) osmotic stabilizer concentration, and (3) digestion duration—we determined that digestion of young leaves for 6 h in darkness using an enzyme solution containing 1.0% (*w*/*v*) Cellulase R-10, 0.7% (*w*/*v*) Macerozyme R-10, and 0.4 M mannitol yielded protoplasts with both high productivity (3.3 × 10^6^ protoplasts g^−1^ FW) and viability (90%).

Furthermore, we validated the feasibility of PEG-mediated transient transformation in Welsh onion protoplasts. While this preliminary investigation confirmed successful GFP expression, future studies should focus on optimizing transformation efficiency through systematic parameter testing (PEG concentration, plasmid DNA amount, and incubation conditions).

The established transient transformation systems significantly advance functional genomics in Allium crops by enabling rapid gene validation (e.g., disease resistance screening within 4–6 weeks versus 6–12 months for stable lines). These protocols are readily transferable to economically vital horticultural species like garlic and leek, where genetic transformation remains challenging. For crop improvement, the systems accelerate CRISPR/Cas target pre-screening and metabolic engineering of high-value compounds (e.g., allicin). In agricultural biotechnology, they provide a scalable platform for real-time pathogen response studies and synthetic pathway assembly—ultimately expediting the development of climate-resilient Allium varieties.

## Figures and Tables

**Table 1:** Result of orthogonal test on induction conditions for hairy roots.

Treatment (No.)	OD_600_	AS (µM)	Induction Rate (%)
1	0.3	100	88.75
2	0.3	200	61.25
3	0.3	300	62.5
4	0.5	100	87.5
5	0.5	200	66.25
6	0.5	300	53.75
7	0.7	100	47.5
8	0.7	200	42.5
9	0.7	300	43.75

**Table 2:** Results of orthogonal test on protoplast separation conditions of Welsh onion leaves.

Treatment No.	Cellulase R-10 (%)	Macerozyme R-10 (%)	Protoplast Yield (×10^5^ Protoplasts·g^−1^ FW)	Protoplast Vitality (%)
1	0.5	0.3	3.33	91.67
2	0.5	0.5	6.33	89.42
3	0.5	0.7	10.56	88.38
4	1.0	0.3	6.56	88.67
5	1.0	0.5	14.33	86.88
6	1.0	0.7	18.44	89.67
7	1.5	0.3	3.11	94.44
8	1.5	0.5	9.00	91.48
9	1.5	0.7	12.00	87.94
*K*_1_	20.22	13.00		
*K*_2_	39.33	29.67		
*K*_3_	24.11	41.00		
*k*_1_	6.74	4.33		
*k*_2_	13.11	9.89		
*k*_3_	8.04	13.67		
*R*	6.37	9.33		
*K*_1_′	269.47	274.78		
*K*_2_′	265.21	267.78		
*K*_3_′	273.86	265.99		
*k*_1_′	89.82	91.59		
*k*_2_′	88.40	89.26		
*k*_3_′	91.29	88.66		
*R*′	2.88	2.93		

Notes: (1) *K_i_*: sum of protoplast production of all experimental groups at a specific factor level of *i*, *k_i_*: mean value of protoplast production at a specific factor level of *i*. *R*: range of protoplast yield. (2) *K_i_*′: sum of protoplast production of all experimental groups at a specific factor level of *i*, *k_i_*′: mean value of protoplast production at a specific factor level of *i*. *R*′: range of protoplast vitality.

## Data Availability

All data points generated or analyzed in this study are included in this paper, and no further underlying data were needed to reproduce the results. Upon reasonable request, we can obtain the original data from the corresponding authors.
